# Supplementary material for: PD-1 pathway regulates ILC2 metabolism and PD-1 agonist treatment ameliorates airway hyperreactivity
Source: Nat Commun. 2020 Aug 10;11:3998. doi: 10.1038/s41467-020-17813-1 (PMC7417739; doi:10.1038/s41467-020-17813-1)
Supplement: Supplementary file 1 — Supplementary Information [file 41467_2020_17813_MOESM1_ESM.pdf]

PD-1 pathway regulates ILC2 metabolism and PD-1 agonist treatment ameliorates airway hyperreactivity

Helou et al., Supplementary information.

Supplementary Table 1

	Total Counts	P value	Fold change (KO vs. WT)
Arl2bp	294.02	3.03E-13	16.19
Fam177a	707.24	6.13E-12	-3.77
Srp54b	453.62	1.54E-11	-3.54
Ly6a	1756.74	2.23E-11	-7.08
Pcna-ps2	241.23	6.41E-11	-17.32
Stk25	458.44	1.10E-10	3.57
Gsn	988.71	5.56E-10	2.73
Ramp1	599.98	7.84E-10	-2.59
Ptges	119.83	8.21E-10	8.85
Tgtp2	346.12	8.28E-10	-3.08
Srp54a	1592.39	1.21E-09	-2.88
Sfi1	558.38	1.43E-09	-3.86
Slc4a7	765.99	1.76E-09	2.31
Rgs1	1058.02	2.07E-09	-2.71
Anxa2	1706.07	2.41E-09	2.05
Ifi47	650.54	2.44E-09	-2.39
Tfe3	490.37	2.80E-09	2.36
AW112010	1484.80	3.05E-09	-2.44
Calcrl	439.77	3.05E-09	2.23
Rtf2	987.39	3.17E-09	2.01
Il6	354.00	6.83E-09	2.63
Itpripl1	187.61	7.11E-09	3.65
Anxa5	1109.07	9.40E-09	1.98
Vegfc	206.05	1.06E-08	3.04
Ighm	138.31	1.15E-08	44.33
Ier3	153.93	1.34E-08	4.24
Ltb	732.60	1.84E-08	-2.28
Myadm	453.18	2.32E-08	2.73
Glo1	1229.23	3.20E-08	-1.93
Plxdc2	161.21	3.75E-08	2.38
Arid5a	1128.86	5.35E-08	-1.97
Itgb3	473.31	6.99E-08	-1.97
Ctla2a	1852.06	8.55E-08	-1.77
Adora2b	233.35	9.08E-08	2.16
Il17rb	146.27	9.26E-08	-2.53
P4ha2	345.71	9.30E-08	1.99
Il13	3509.04	1.23E-07	1.97
Gimap4	190.46	1.28E-07	-2.77
Phlda1	378.26	1.46E-07	2.34
Atp8b4	159.92	1.47E-07	2.18
Hspa1b	181.61	1.52E-07	2.83
Neurl3	450.51	1.59E-07	-2.42
Psmb9	647.80	1.79E-07	-2.03
Emilin2	219.19	1.87E-07	2.20
Mindy1	513.26	1.87E-07	1.72
Upp1	811.00	1.90E-07	1.88
Igtp	1060.68	2.17E-07	-1.99
Lilrb4a	540.84	2.22E-07	2.41
Csf2	10784.59	2.23E-07	2.18
Traf1	2757.31	2.45E-07	1.68

Top 50 differentially regulated genes

ILC2s were sorted from WT and PD-1 KO mice after 3 intranasal challenges with 0.5 µg of rm-IL-33 and defined as activated (aILC2s). Sorted cells were incubated with rm-IL-2 (10 ng.mL^{-1}) and rm-IL-7 (10 ng.mL^{-1}) for 24 h.

Genes are ranked according to significance in differential expression (PD-1 KO vs. WT aILC2s; n=3; total counts >100).

<u>Supplementary Figure 1</u>

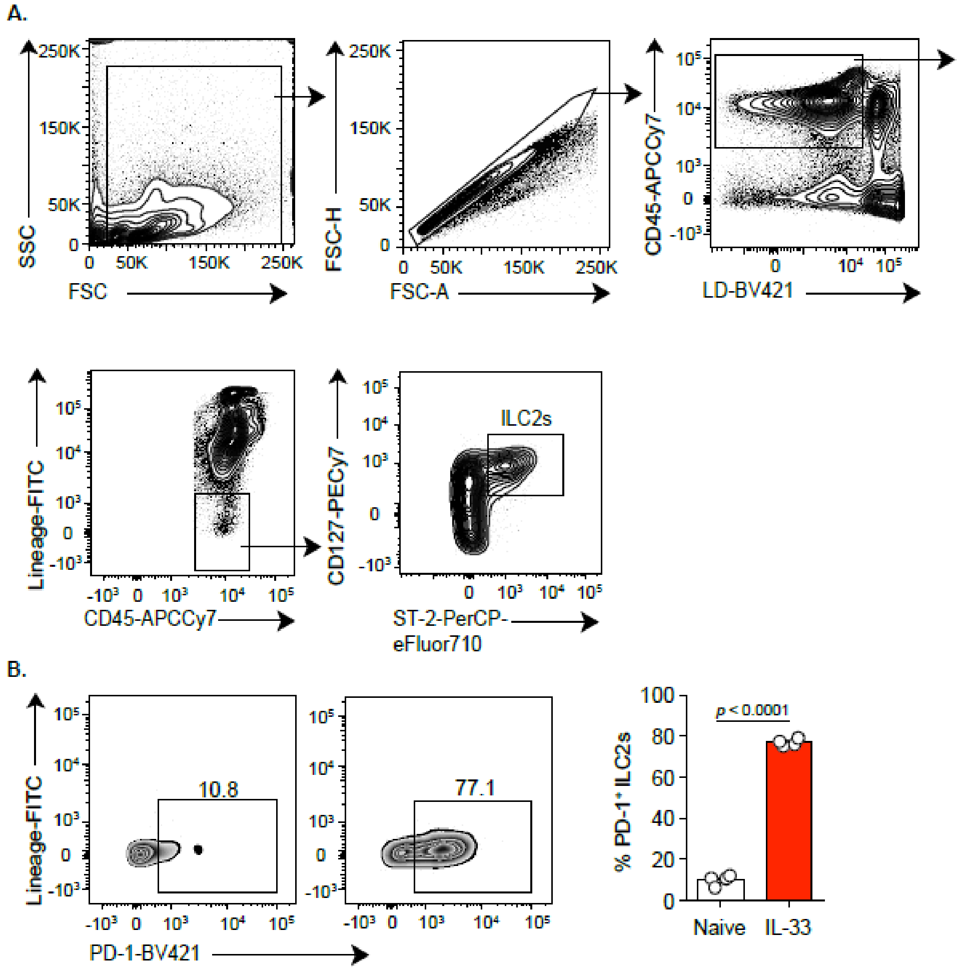

PD-1 is expressed on a small subset of pulmonary ILC2s at steady state.

(A) Full gating strategy of pulmonary ILC2s defined by a lack of lineage markers (CD3e, CD45R, Gr-1, CD11c, CD11b, Ter119, NK1.1, TCR-γδ, TCR-β, CD5 and FcεRI) and expression of CD45, ST2 and CD127. Dead cells were excluded using a life dead staining.

(B) BALB/cByJ mice (WT) mice were challenged or not (naïve) with 0.5 µg of rm-IL-33 for 3 consecutive days. Representative flow cytometry plots and corresponding quantification of the percentage of PD-1$^+$ ILC2s (right).

Data are presented as means ± SEM (n=4; two-tailed Student's t-test).

<u>Supplementary Figure 2</u>

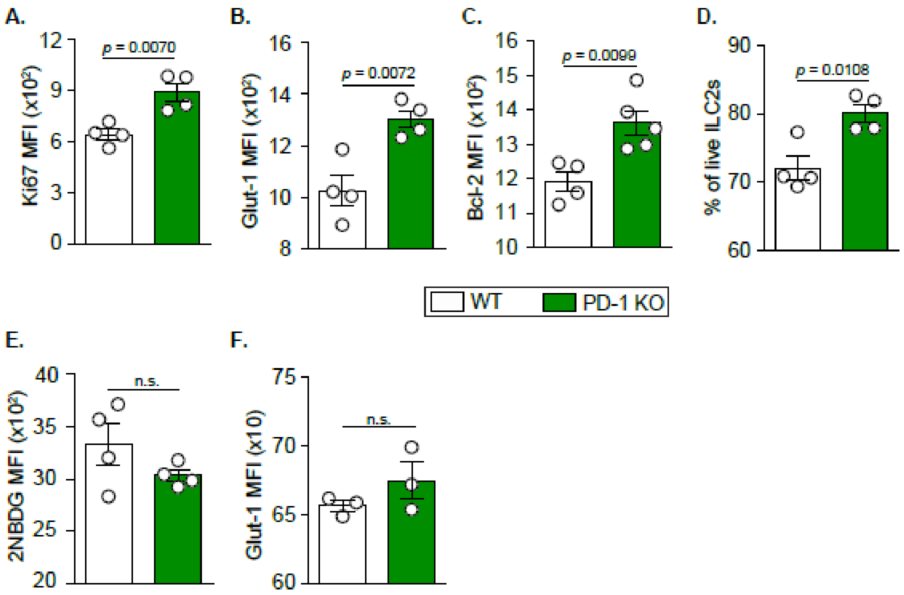

PD-1 decreases the survival and proliferation of *in vitro*-activated ILC2s

(A-D) ILC2s were sorted from naïve WT and PD-1 KO mice and cultured for 48 h in the presence of rm-IL-2 (10 ng.mL^{-1}), rm-IL-7 (10 ng.mL^{-1}) and IL-33 (20 ng.mL^{-1}).

(A) Quantification of ki67, (B) Glut-1 and (C) Bcl-2 presented as MFI; n=4.

(D) Percentage of live ILC2s after 48 h of *in vitro* stimulation; n=4.

(E) Quantification of 2NDBG uptake (n=4) and (F) Glut-1 expression presented as MFI in WT and PD-1 KO freshly sorted ILC2s; n=3.

Data are presented as means ± SEM (two-tailed Student's t-test, n.s.: non-significant).

Supplementary Figure 3

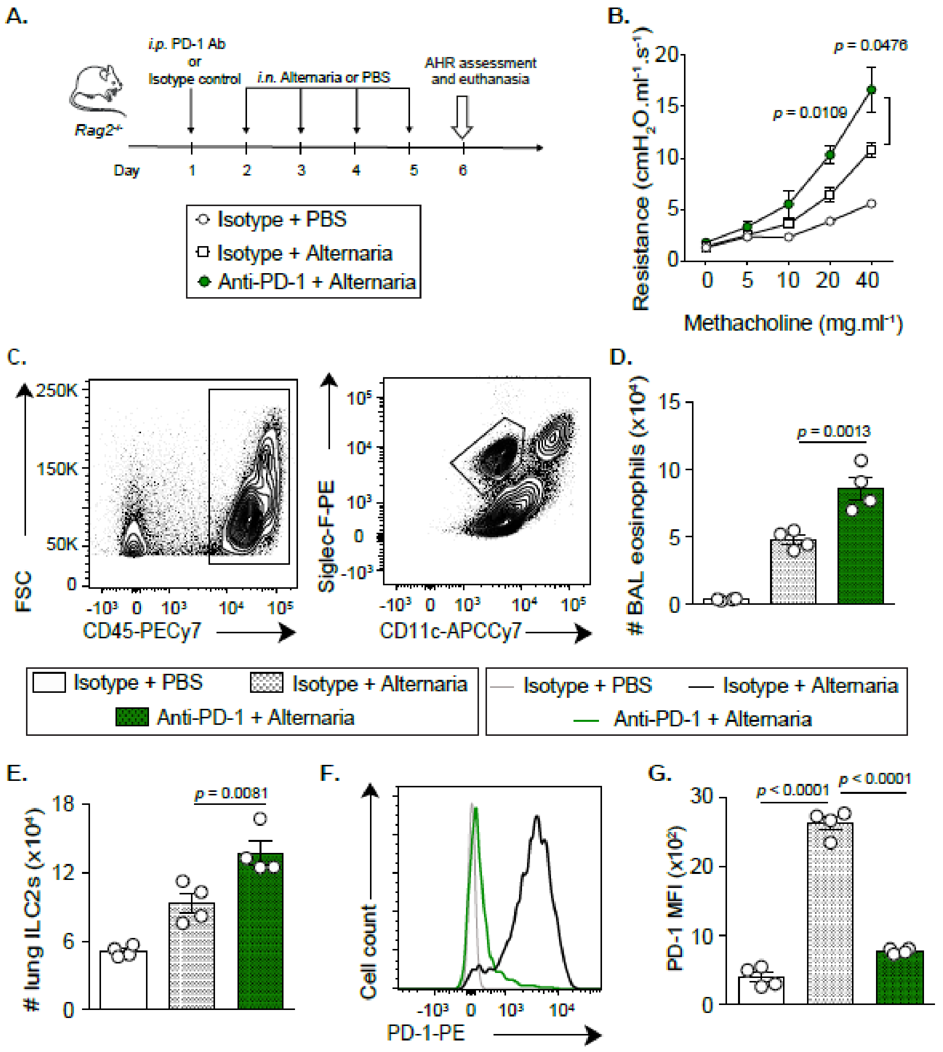

PD-1 downregulates AHR and lung inflammation in Alternaria model

(A-G) *Rag2*$^{-/-}$ mice received intraperitoneal injection (i.p.) of anti-PD-1 blocking antibody (500 µg) or isotype control at day 1. Then mice were challenged intranasally from day 2 to 5 with an extract of *Alternaria alternata* (100 µg). On day 6, AHR and lung inflammation were assessed.

(B) Lung resistance in response to increasing concentrations of methacholine.

(C) Eosinophils in the BAL gated as CD45$^+$ SiglecF$^+$ CD11c$^-$.

(D) Total number of eosinophils in BAL and (E) total number of pulmonary ILC2s.

(F) Representative histogram of PD-1 expression on pulmonary ILC2s and (G) corresponding MFI quantification.

Data are presented as means ± SEM (n=4; two-tailed Student's t-test or 1-way ANOVA). Mouse image provided with permission from Servier Medical Art.

Supplementary Figure 4

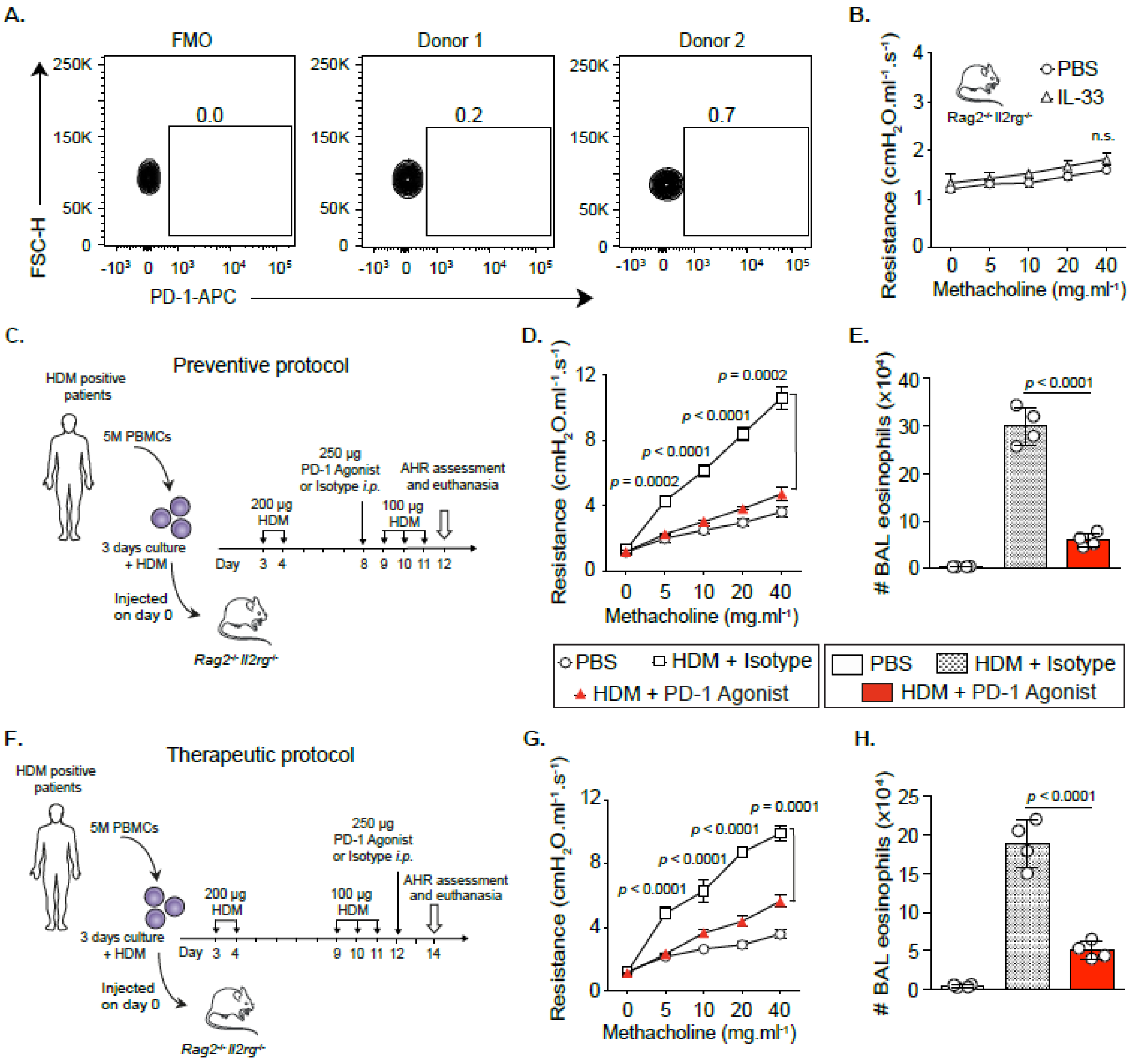

PD-1 agonist demonstrates a therapeutic and preventive efficacy in IL-33 and HDM-induced asthma models

(A) Flow cytometry plots representing the expression of PD-1 on freshly sorted ILC2s from 2 healthy donors.

(B) *Rag2*$^{-/-}$ *Il2rg*$^{-/-}$ mice were challenged with 1 μg of rh-IL-33 for 3 consecutive days. No adoptive transfers were performed. Lung resistance measured in restrained tracheostomized mechanically ventilated mice, n=3.

(C-E) Total PBMCs from HDM positive patients were adoptively intravenously transferred into *Rag2*$^{-/-}$ *Il2rg*$^{-/-}$ mice. Intranasal challenges were performed with HDM or PBS on days 3, 4, 9, 10 and 11. At day 8, mice received an intraperitoneal injection of PD-1 agonist or control isotype (250 μg). Measurement of lung function and inflammation were performed on day 12.

(D) Lung resistance measured in restrained tracheostomized mechanically ventilated mice exposed to increasing concentrations of methacholine.

(E) Total number of eosinophils in BAL gated as $CD45^+$ $SiglecF^+$ $CD11c^-$.

(F-H) Total PBMCs from HDM positive patients were adoptively intravenously transferred into *Rag2*$^{-/-}$ *Il2rg*$^{-/-}$ mice. Intranasal challenges were performed with HDM or PBS on days 3, 4, 9, 10 and 11. At day 12, mice received an intraperitoneal injection of PD-1 agonist or control isotype (250 μg). Measurement of lung function and inflammation were performed on day 14.

(G) Lung resistance measured in restrained tracheostomized mechanically ventilated mice exposed to increasing concentrations of methacholine.

(H) Total number of eosinophils in BAL gated as $CD45^+$ $SiglecF^+$ $CD11c^-$.

Data are presented as means ± SEM (n=4; two-tailed Student's t-test or 1-way ANOVA). Mouse and human images provided with permission from Servier Medical Art.
