# Supplementary material for: PD-1 pathway regulates ILC2 metabolism and PD-1 agonist treatment ameliorates airway hyperreactivity
Source: Nat Commun. 2020 Aug 10;11:3998. doi: 10.1038/s41467-020-17813-1 (PMC7417739; doi:10.1038/s41467-020-17813-1)
Supplement: Supplementary file 3 — Reporting Summary [file 41467_2020_17813_MOESM3_ESM.pdf]

nature research

Corresponding author(s): Omid Akbari

Last updated by author(s): 07/09/2020

Reporting Summary

Nature Research wishes to improve the reproducibility of the work that we publish. This form provides structure for consistency and transparency in reporting. For further information on Nature Research policies, see our Editorial Policies and the Editorial Policy Checklist.

Statistics

For all statistical analyses, confirm that the following items are present in the figure legend, table legend, main text, or Methods section.

n/a	Confirmed	
☐	☒	The exact sample size (*n*) for each experimental group/condition, given as a discrete number and unit of measurement
☐	☒	A statement on whether measurements were taken from distinct samples or whether the same sample was measured repeatedly
☐	☒	The statistical test(s) used AND whether they are one- or two-sided<br>*Only common tests should be described solely by name; describe more complex techniques in the Methods section.*
☒	☐	A description of all covariates tested
☒	☐	A description of any assumptions or corrections, such as tests of normality and adjustment for multiple comparisons
☐	☒	A full description of the statistical parameters including central tendency (e.g. means) or other basic estimates (e.g. regression coefficient) AND variation (e.g. standard deviation) or associated estimates of uncertainty (e.g. confidence intervals)
☐	☒	For null hypothesis testing, the test statistic (e.g. *F*, *t*, *r*) with confidence intervals, effect sizes, degrees of freedom and *P* value noted<br>*Give P values as exact values whenever suitable.*
☒	☐	For Bayesian analysis, information on the choice of priors and Markov chain Monte Carlo settings
☒	☐	For hierarchical and complex designs, identification of the appropriate level for tests and full reporting of outcomes
☒	☐	Estimates of effect sizes (e.g. Cohen's *d*, Pearson's *r*), indicating how they were calculated

Our web collection on statistics for biologists contains articles on many of the points above.

Software and code

Policy information about availability of computer code

Data collection	BD FACSDiva software v8.0.1 was used for flow cytometry data aquisition
Data analysis	Partek Genomics Suite software, version 7.0 Copyright, FlowJo software (TreeStar) version 10, LEGENDplex data analysis software v8.0, ImageJ , Prism analysis software version 5-8

For manuscripts utilizing custom algorithms or software that are central to the research but not yet described in published literature, software must be made available to editors and reviewers. We strongly encourage code deposition in a community repository (e.g. GitHub). See the Nature Research guidelines for submitting code & software for further information.

Data

Policy information about availability of data

All manuscripts must include a data availability statement. This statement should provide the following information, where applicable:

- Accession codes, unique identifiers, or web links for publicly available datasets
- A list of figures that have associated raw data
- A description of any restrictions on data availability

Transcriptional data that support the findings of this study have been deposited in Genbank with the primary accession code [GSE153909]. All remaining data will be made available by the corresponding author upon reasonable request.

Field-specific reporting

Please select the one below that is the best fit for your research. If you are not sure, read the appropriate sections before making your selection.

- [x] Life sciences
- [ ] Behavioural & social sciences
- [ ] Ecological, evolutionary & environmental sciences

For a reference copy of the document with all sections, see nature.com/documents/nr-reporting-summary-flat.pdf

Life sciences study design

All studies must disclose on these points even when the disclosure is negative.

Sample size	We used ANOVA to calculate the sample size with the assumption of power of 80%, effect size of ~~50, and alpha less than 0.05.
Data exclusions	No animals were excluded. There was no anticipation of excluding animals once they underwent the experiments.
Replication	All attempts at replication were successful. Data are representative of three or at least two independent experiments.
Randomization	In all experiments, age- and sex- matched mice were randomly allocated to experimental groups. Randomization process consisted of pooling mice from the same genotype in a large cage and random picking out and allocating to experimental groups.
Blinding	For some experiments including AHR measurements and histological analysis, blinding was applied in order to avoid unconscious bias. No blinding was done for the remaining experiments as the design and conditions prevent possible sources of bias.

Reporting for specific materials, systems and methods

We require information from authors about some types of materials, experimental systems and methods used in many studies. Here, indicate whether each material, system or method listed is relevant to your study. If you are not sure if a list item applies to your research, read the appropriate section before selecting a response.

Materials & experimental systems

n/a	Involved in the study	
[ ]	[x]	Antibodies
[x]	[ ]	Eukaryotic cell lines
[x]	[ ]	Palaeontology and archaeology
[ ]	[x]	Animals and other organisms
[ ]	[x]	Human research participants
[x]	[ ]	Clinical data
[x]	[ ]	Dual use research of concern

Methods

n/a	Involved in the study	
[x]	[ ]	ChIP-seq
[ ]	[x]	Flow cytometry
[x]	[ ]	MRI-based neuroimaging

Antibodies

Antibodies used

Anti-mouse antibodies
Biotin - CD3e (supplier: BioLegend; cat. #: 100304; clone: 145-2C11; lot #: B216147)
Biotin - CD45R/B220 (supplier: BioLegend; cat. #: 103204; clone: RA3-6B2; lot #: B226660)
Biotin - Gr-1 (supplier: BioLegend; cat. #: 108404; clone: RB6-8C5; lot #: B200655)
Biotin - CD11c (supplier: BioLegend; cat. #: 117304; clone: N418; lot #: B217819)
Biotin - CD11b (supplier: BioLegend; cat. #: 101204; clone: M1/70; lot #: B221905)
Biotin - Ter119 (supplier: BioLegend; cat. #: 116204; clone: TER-119; lot #: B218645)
Biotin - FceRIa (supplier: BioLegend; cat. #: 134304; clone: MAR-1; lot #: B218942)
Biotin - CD5 (supplier: BioLegend; cat. #: 1000604; clone: 53-7.3; lot #: B202966)
Biotin - TCR-gd (supplier: eBioscience; cat. #: 13-5711-85; clone: eBioGL3; lot #: 4274982)
Biotin - TCR-b (supplier: BioLegend; cat.# 109204; clone: H57-597; lot #: B279070)
FITC - Streptavidin (supplier: BioLegend; cat. #: 405202; lot #: B190908)
PE-Cy7 - CD127 (supplier: BioLegend; cat. #: 135014; clone: A7R34; lot #: B222850)
APC - CD127 (supplier: BioLegend; cat.#: 135012; clone A7R34; lot #: B181517)
APCCy7 - CD45 (supplier: BioLegend; cat. #: 103116; clone: 30-F11; lot #: B237400)
PerCP - eFluor710-ST2 (supplier: eBioscience; cat. #: 46-9335-82; clone: RMST2-2; lot #: 4300549)
APC/Cy7 - CD11c (supplier: BioLegend; cat. #: 117324; clone: N418; lot #: B237079)
PerCP Cy5.5- CD11c (supplier: BioLegend; cat. #: 117327; clone: N418; lot #: B256449)
BV421-CD11b (supplier: BD Biosciences; cat 560455, clone:M1/70, lot #: 31294)
APC-Gr1 (supplier: BioLegend; cat.#: 108412, clone: RB6-8C5, lot #: B236640)
PECy7 - CD45 (supplier: BioLegend; cat. #: 103114; clone: 30-F11; lot #: B219150)

	PE-Siglec-F (supplier BD Bioscеinces; cat.#: 552126; clone: E50-2440; lot #:5343971)<br>BV421 -PD-1 (supplier BD Biosciences; cat. #: 562584; clone: J43; lot #: 4352992)<br>PE- PD-1: (supplier eBioscience ; cat.#: 12-9985-82; clone: J43; lot: E02309-187)<br>APC - PD-L1: (supplier BioLegend, cat.#124312; clone: 10F.9G2, lot #: B204732)<br>PECy7-PD-L1: (supplier BioLegend, cat.#124314; clone: 10F.9G2, lot #: B165159)<br>PE - PD-L2: (supplier BioLegend, cat.#107205; clone: TY25, lot #: B257232)<br>PE - IL-5 (supplier: BioLegend; cat. #: 504304; clone: TRFK5; lot #: B204095)<br>eFluor 450 - IL-13 (supplier: eBioscience; cat. #: 48-7133-82; clone: eBio13A; lot #: 4318966)<br>PE-Cy7 Glut-1 (supplier: Novus Biologicals; cat.#: NB110-38113; polyclonal; lot #: G-3-042420-PECY7)<br>PE-Cy7- Bcl-2 (supplier: eBiosciences; cat.#: 25-6992-42; clone: 10C4; lot#:25-6992-42)<br>PE- GATA-3 (supplier: invitrogen; cat.#: 12-9966-42; clone: TWAJ; lot#: 2075111)<br>APC- Ki67((supplier: invitrogen; cat.#: 50-5698-82; clone: SolA15; lot#: 2085694)<br>anti-mouse PD-1 (CD279) blocking antibody (supplier: BioXcell; clone: 29F.1A12; cat#:BE0273)<br>rat IgG2a isotype control (supplier: BioXcell; clone: 2A3, cat#: BE0089, Lot #: 673018A2)<br><br>Anti-human antibodies<br>FITC - Lineage (supplier: BioLegend; cat. #: 348801; lot #: B215228)<br>FITC - CD235a (supplier: BioLegend; cat. #: 349104; clone: HI264; lot #: B251984)<br>FITC - FCeRIa (supplier: BioLegend; cat. #: 334608; clone: AER-37; lot #: B226717)<br>FITC - CD1a (supplier: BioLegend; cat. #: 300104; clone: HI149; lot #: B155264)<br>FITC - CD123 (supplier: BioLegend; cat. #: 306014; clone: 6H6; lot #: B167088)<br>APCCy7 - CD45 (supplier: BioLegend; cat. #: 304014; clone: HI30; lot #: B214034)<br>PE - CD294 (CRTH2) (supplier: BioLegend; cat. #: 350106; clone: BM16; lot #: B179655)<br>PE/Cy7 - CD127 (IL-7Ra) (supplier: BioLegend; cat. #: 351320; clone: A019D5; lot #: B191896)<br>APC - CD279 (PD-1) (supplier: BioLegend; cat. #329908, clone EH12.2H7; lot #: B254071)<br>Human PD-1 agonist and corresponding isotype (supplier Janssen Pharmaceuticals; patent WO 2018/226580 A2)
Validation	All the antibodies are from commercial source and have been validated by the vendors and their validation data are available on the manufacturers' website (BioLegend, BioXcell, Novus Biologicals, invitrogen, BD Biosciences and eBioscience). Human PD-1 agonist was developed and validated by Janssen Pharmaceuticals, patent WO 2018/226580 A2)

Animals and other organisms

Policy information about studies involving animals; ARRIVE guidelines recommended for reporting animal research

Laboratory animals	We used male 6-8 week mice. Strains include: BALB/cBYJ (JAX:001026); PD-1 Knockout mice generated and provided by Sharpe Laboratory, Rag2- gc- (JAX: 014593); Rag2 KO (JAX: 008449).<br>Mice were maintained at macroenvironmental temperature of 21-22 °C, humidity (48-52%), in a conventional 12:12 light/dark cycle with lights on at 6:00 a.m. and off at 6:00 p.m.
Wild animals	No wild animals were used in this study.
Field-collected samples	No field-collected samples were used in this study.
Ethics oversight	All the experiments described in this manuscript were approved IACUC of USC and in complete compliance with the guidelines of IACUC of USC. Animal facilities at the USC are AAALAC accredited.

Note that full information on the approval of the study protocol must also be provided in the manuscript.

Human research participants

Policy information about studies involving human research participants

Population characteristics	Human blood samples were obtained from male and female healthy donors (Age 18 to 65).<br>In few experiments, blood was collected from allergic subjects having positive skin tests to house dust mite antigen. Informed consent was obtained from all donors according to our approved IRB protocols.
Recruitment	No specific selection was applied for healthy donors. For the recruitment of allergic patients, only HDM positive patients with allergic asthma were selected according to our approved IRB protocols.
Ethics oversight	All human studies were approved by USC Institutional review board and conducted in accordance to the principles of the Declaration of Helsinki.

Note that full information on the approval of the study protocol must also be provided in the manuscript.

Flow Cytometry

Plots

Confirm that:

- [x] The axis labels state the marker and fluorochrome used (e.g. CD4-FITC).
- [x] The axis scales are clearly visible. Include numbers along axes only for bottom left plot of group (a 'group' is an analysis of identical markers).
- [x] All plots are contour plots with outliers or pseudocolor plots.
- [x] A numerical value for number of cells or percentage (with statistics) is provided.

Methodology

Sample preparation	This information is included in the Method section, in the Tissue preparation and Flow Cytometry subsection.
Instrument	Stained cells were analyzed on FACSCanto II and/or FACSARIA III systems (Becton Dickinson)
Software	The software used to collect samples is BD FACSDiva software v8.0.1 and the data were analyzed with FlowJo version 10 software (TreeStar, Ashland, Oregon)
Cell population abundance	10 000 to 20 000 naïve ILC2s were sorted per mouse. Purity was assessed by analyzing sorted cells for the same markers used for sorting. Purity greater than or equal to 90% was considered satisfactory.
Gating strategy	Relevant gating strategies shown in Figure 1, 2, 5, 7 and sup Fig 1, 3, 4.

- [x] Tick this box to confirm that a figure exemplifying the gating strategy is provided in the Supplementary Information.
